# An Efficient Beam Element Based on Quasi-3D Theory for Static Bending Analysis of Functionally Graded Beams

**DOI:** 10.3390/ma12132198

**Published:** 2019-07-08

**Authors:** Hoang Nam Nguyen, Tran Thi Hong, Pham Van Vinh, Do Van Thom

**Affiliations:** 1 Modeling Evolutionary Algorithms Simulation and Artificial Intelligence, Faculty of Electrical & Electronics Engineering, Ton Duc Thang University, Ho Chi Minh City 700000, Vietnam; nguyenhoangnam@tdtu.edu.vn; 2 Center of Excellence for Automation and Precision Mechanical Engineering, Nguyen Tat Thanh University, Ho Chi Minh City 700000, Vietnam; hongtt@ntt.edu.vn; 3 Department of Mechanics, Le Quy Don Technical University, Hanoi City 100000, Vietnam; phamvanvinh@lqdtu.edu.vn

**Keywords:** beam element, Quasi-3D, static bending, functionally graded beam

## Abstract

In this paper, a 2-node beam element is developed based on Quasi-3D beam theory and mixed formulation for static bending of functionally graded (FG) beams. The transverse shear strains and stresses of the proposed beam element are parabolic distributions through the thickness of the beam and the transverse shear stresses on the top and bottom surfaces of the beam vanish. The proposed beam element is free of shear-looking without selective or reduced integration. The material properties of the functionally graded beam are assumed to vary according to the power-law index of the volume fraction of the constituents through the thickness of the beam. The numerical results of this study are compared with published results to illustrate the accuracy and convenience rate of the new beam element. The influence of some parametrics on the bending behavior of FGM beams is investigated.

## 1. Introduction

Functionally graded (FG) materials (FGM) are one of the advanced composites. In which the material properties of FGM vary continuously through one or more directions. A typical, the material properties of an FGM beam, plate and shell varies continuously through the thickness direction. Due to their advantages, the FGMs have used widely in many fields such as civil, aerospace, automobile, engineering, nuclear power plants and so on [1,2]. Since then, many scientists have been focused on the mechanical analysis of FG beams, plates and shells. In which, they used several theories and methods, for instance, analytical and numerical methods based on Euler-Bernoulli theory, Timoshenko theory or first-order shear deformation theory (FSDT), higher-order shear deformation theory (HSDT), Quasi-3D theory and Carrera Unified Formulation (CUF).

Sankar [3] developed an elasticity solution to analyze a simply supported FG beam subjected to transverse distribution loading. In his work, Sankar developed new beam theory which was similar to the Euler-Bernoulli beam theory. Zenkour [4] analyzed an exponentially graded thick rectangular plate using both 2-D and 3-D elasticity solutions. Zhong et al. [5] analyzed a cantilever FG beam using a general two-dimensional solution. The free vibration and buckling analysis of FG beams under mechanical and thermal loads were investigated by Trinh et al. [6] using the analytical method based on the state space approach and higher-order beam theory.

The Euler-Bernoulli beam theory ignores the shear deformation so that it provides an acceptable solution for thin beams only; this model requires a $C^{1}$ continuity for a compatible displacement formulation. Kien [7] used the Euler-Bernoulli beam model to analyze large displacement behaviors of tapered cantilever FG beams by the finite element method. Lee et al. [8] applied Euler-Bernoulli beam theory for free vibration analysis of FG beam using an exact transfer matrix expression.

Due to the shear deformation effects are more obvious in thick beams and plates, so the FSDT can be used in these cases. On the other hand, it requires only $C^{0}$ continuity for the deflection. One of the shortcomings of the FSDT is the transverse shear strain distributes in an inaccurate and it does not satisfy the stress-free boundary conditions at the top and bottom surfaces of the structures, so this model requires a shear correction factor. Menaa et al. [9] used the energy equivalence principle to derive a general expression for the static shear correction coefficients in FG beams. Modal analysis of FG beams with shear correction function was studied by Murin et al. [10]. Nguyen et al. [11] employed FSDT for static and free vibration of axially loaded FG beams. Nam et al. [12] investigated the mechanical behaviors of variable thickness FG beam using modified FSDT. Due to the simplicity and effectiveness of FSDT, many scientists have applied FSDT to analyze plates and beams, and it is still being improved to achieve higher accuracy.

The shear correction coefficients can be removed by using HSDT, which have been developed by many scientists. In this model, the transverse shear strain varies parabolically through the height of the structures, and the transverse shear stresses at the top and bottom surfaces of the structures are neglected so that it need not any shear correction coefficients. Shi [13] proposed a new simple third-order shear deformation theory (TSDT) to analyze static bending of rectangular plates. Kadoli et al. [14] used HSDT for static bending analysis of FG beams. Benatta et al. [15] studied static bending of FG short beam involving the effects of warping and shear deformation. Li et al. [16] investigated static bending and dynamic response of FG beams using HSDT. Thai et al. [17] applied various HSDT to analyze static bending and free vibration of FG beams. Refined shear deformation was applied for static bending and vibration analysis of FG beams by Vo et al. [18]. Tinh et al. [19] used finite element method (FEM) and a new TSDT for mechanical response analysis of heated FGM plates.

Both FSDT and HSDT ignore the effect of the thickness stretching, which is noticeable in thick beams and plates. A number of Quasi-3D theories have been developed, in which the effects of shear deformation and thickness stretching were included. Vo et al. [20] used a Quasi-3D theory with only four unknown components to investigate the static behavior of FG beams and FG sandwich beams. Neves et al. [21,22] and Hebali [23] developed a Quasi-3D theory with sinusoidal shear function and hyperbolic shear deformation theory to analyze the static bending and free vibration of FG plates. Mantari et al. [24] studied static bending of advanced composite plates using a generalized hybrid Quasi-3D shear deformation theory. Mantari et al. [25] used a four-unknown Quasi-3D shear deformation theory for analysis of advanced composite plates. Thai et al. [26] presented a Quasi-3D hyperbolic shear deformation theory for analysis FG plates. Fang et al. [27] applied Quasi-3D theory and isogeometric analysis to study thick porous beams. Nguyen et al. [28] and Yu et al. [29] used Quasi-3D theory and isogeometric analysis to investigate FG microplates and two-directional FG microbeams. Farzam-Rad et al. [30] applied Quasi-3D theory and isogeometric analysis to study FG plates based on the physical neutral surface. Tran et al. [31] employed a Quasi-3D model with six-variable for static analysis of laminated composite plate using isogeometric analysis. The most outstanding of Quasi-3D theory is applicable to analyze thick plates and beams where the normal deformation effect is significant.

Carrera [32] developed Unified Formulation (CUF), which produces any refined theories for many structures such as beams, plates, and shells. Cerrera et al. [33] applied CUF for free vibration finite element analysis of beams with a uniform section. Cerrera et al. [34] employed CUF for studying micropolar beams using an analytical method. The 1D CUF theories were applied to analyze FG beams using FEM by Giunta et al. [35] and Filippi et al. [36].

However, in HSDT and Quasi-3D theory, the displacement field is considered by the existence of the higher order derivative of the deflection of transverse. So that it involves the development of a $C^{1}$ continuity element, which can cause difficulty to originate the second derivative of deformation in FEM. To overcome these continuity issues, Hermite interpolation functions with $C^{1}$ elements and some $C^{0}$ approximations have been adopted. Chakraborty et al. [37] developed a new beam element based on the FSDT and an exact solution of the static part of the governing differential equations for analysis of FGM structures. Nguyen et al. [38] applied the Timoshenko beam model and FEM for dynamic response of bi-directional FG beams subjected to moving load. Khan et al. [39] investigated the static bending and free vibration of FG beams using FEM. Heyliger [40] developed a higher order beam finite element for bending and vibration of beams. Kapuria et al. [41] studied bending behavior and free vibration response of layered FG beams using a third order zigzag theory and FEM. Based on refined shear deformation theory, Vo et al. [42] developed a finite element model to analyze free vibration and buckling of FG sandwich beams. Moallemi-Oreh et al. [43] used FEM for stability and free vibration analysis of the Timoshenko beam. Pascon [44] analyzed FG beams with variable Poisson’s ratio using FEM. Yarasca et al. [45] studied FG sandwich beams using Hermite-Lagrangian finite element formulation. The use of higher-order shape function will cost much computation effort in comparison with linear shape function. Furthermore, the linear shape function is simpler in formulation and transformation than higher-order shape function. However, plate and beam element using linear shape function are mainly developed based on FSDT and HSDT. To author’s knowledge, there is currently no beam element using linear shape function based on a Quasi-3D theory. Therefore, the development of a beam element using linear shape function based on a Quasi-3D theory is necessary.

This paper presents a new beam element based on Quasi-3D theory, which only requires $C^{0}$ shape functions. The organization of this study is as follows. Firstly, Section 2 defines the model and material properties of FG beams. In Section 3, the governing equations of FG beams based on Quasi-3D theory are given. The finite element formulations of the proposed beam element are presented in Section 4. In Section 5, some example problems are carried out to show the convergence and accurate rate of new beam element in comparison with published data. Then, the static bending behaviors of FG beams using the proposed beam element are studied. The influences of the distribution of materials properties, length-to-thickness ratio, boundary conditions and effect of normal strain are investigated. Finally, in the conclusion section, some remarks on the proposed beam element are given.

## 2. Functionally Graded Material

**Figure 1:**
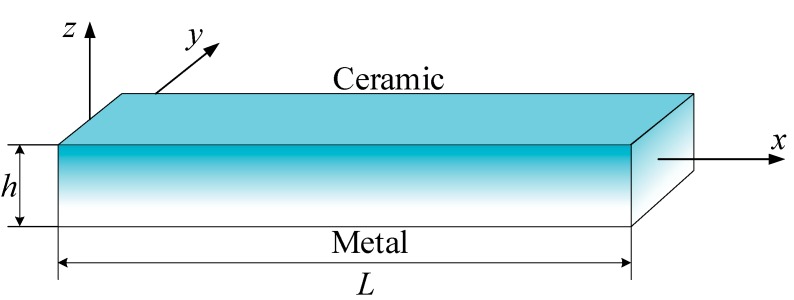
The FG beam model.

Consider an FG beam as shown in Figure 1, the length of the beam is L, the width of the beam is b, and the thickness of the beam is h. The Young’s modulus varies continuously through the thickness of the beams with a power law distribution [19,20]:

(1) $$E\left(z\right)=E_{m}+\left(E_{c}-E_{m}\right){\left(\frac{z}{h}+\frac{1}{2}\right)}^{p}$$

in which subscript m denotes the metallic component and c denotes the ceramic component, $E_{m},\;E_{c}$ are respectively Young’s modulus of the metal and ceramic, p is the power-law index. In this study, the Poisson’s ratios ν of both components are assumed to be constant and equal.

## 3. Governing Equations

The displacements of a point of the beam are expressed by

(2) $$\left\{ \begin{array}{l} u(x,z)=u(x)+f_{1}(z)\beta (x)+f_{2}(z)\alpha (x)\\ w(x,z)=w(x)+g(z)\varphi (x) \end{array}\right.$$

The functions $f_{1}(z),\;f_{2}(z)$ are given by Shi [13]

(3) $$f_{1}(z)=\frac{5}{4}\left(z-\frac{4z^{3}}{3h^{2}}\right);\;f_{2}(z)=-\frac{z}{4}+\frac{5z^{3}}{3h^{2}};\;g(z)={f^{\prime }}_{1}(z)$$

The strain field is obtained as follows

(4) $$\left\{ \begin{array}{l} \varepsilon_{x}=u_{,x}+f_{1}(z)\beta_{,x}+f_{2}(z)\alpha_{,x}\\ \varepsilon_{z}=g^{\prime }(z)\varphi \\ \gamma_{xz}=w_{,x}+f_{1}\prime \varphi_{,x}+f_{1}\prime (z)\beta +f_{2}\prime (z)\alpha \end{array}\right.$$

in which the symbol (,) means the derivatives with respect to the quantity following it and the symbol (′) means the derivatives with respect to z direction.

Rewrite the strain components in the short form as follows

(5) $$\varepsilon =\varepsilon^{0}+f_{1}\varepsilon^{1}+f_{2}\varepsilon^{2}+g^{\prime }\varepsilon^{3}$$

where

(6) $$\varepsilon^{0}=\left\{\begin{array}{l} u_{,x}\\ 0 \end{array}\right\},\;\varepsilon^{1}=\left\{\begin{array}{l} \beta_{,x}\\ 0 \end{array}\right\},\;\varepsilon^{2}=\left\{\begin{array}{l} \alpha_{,x}\\ 0 \end{array}\right\},\;\varepsilon^{3}=\left\{\begin{array}{l} 0\\ \varphi \end{array}\right\}$$

Rewrite the transverse shear strain $\gamma_{xz}$ as follows

(7) $$\gamma_{xz}=f_{1}\prime (\gamma_{0}+\gamma_{1})+f_{2}\prime \gamma_{2}\;,\;\gamma_{0}=\varphi_{,x}\;,\;\gamma_{1}=w_{,x}+\beta \;,\;\gamma_{2}=w_{,x}+\alpha$$

The transverse shear strain is assumed to have a quadratic distribution across the thickness of the beam. In addition, the transverse shear strain equals to zero at the top and bottom surfaces of the beam. These conditions lead to

(8) $$\begin{array}{l} \gamma_{2}=w_{,x}+\alpha =0\\ \gamma_{xz}=f_{1}\prime (z)(\gamma_{0}+\gamma_{1}) \end{array}$$

The constitutive relations between the stress field and the strain field are expressed as follows

(9) $$\left\{\begin{array}{l} \sigma_{x}\\ \sigma_{z}\\ \tau_{xz} \end{array}\right\}=\left[\begin{array}{ccc} C_{11} & C_{13} & 0\\ C_{13} & C_{33} & 0\\ 0 & 0 & C_{55} \end{array}\right]\left\{\begin{array}{l} \varepsilon_{x}\\ \varepsilon_{z}\\ \gamma_{xz} \end{array}\right\}$$

In this study, Young’s modulus E of FGM is a function of the coordinate, whereas, the Poisson’s ratio is assumed to be constant and equal, the coefficients $C_{ij}$ vary with the position according to the following formulas [17]

(10) $$C_{11}=C_{33}=\frac{E(z)}{1-\nu^{2}},\;C_{13}=\nu C_{11},\;C_{55}=\frac{E(z)}{2\left(1+\nu \right)}$$

Equation (9) may be rewritten in the short form as

(11) $$\sigma =D\varepsilon =D\left(\varepsilon^{0}+f_{1}\varepsilon^{1}+f_{2}\varepsilon^{2}+g^{\prime }\varepsilon^{3}\right),\;\tau_{xz}={f^{\prime }}_{1}G\gamma_{xz}$$

where

(12) $$D=\frac{E(z)}{1-\nu^{2}}\left[\begin{array}{cc} 1 & \nu \\ \nu & 1 \end{array}\right],\;G=C_{55}=\frac{E(z)}{2\left(1+\nu \right)}$$

## 4. Finite Element Formulation

The expression of the strain energy of the beam is

(13) $$\Pi =\frac{1}{2}\int_{V}\left(\varepsilon^{T}.\sigma +\gamma_{xz}.\tau_{xz}\right)dV$$

The expression of the variation of strain energy can be calculated as follows

(14) $$\delta \Pi =\int_{V}\left\{\begin{array}{l} {\left[\delta \varepsilon^{0}+f_{1}\delta \varepsilon^{1}+f_{2}\delta \varepsilon^{2}+g^{\prime }\delta \varepsilon^{3}\right]}^{T}.D\left(\varepsilon^{0}+f_{1}\varepsilon^{1}+f_{2}\varepsilon^{2}+g^{\prime }\varepsilon^{3}\right)\\ +\delta (\gamma_{0}+\gamma_{1}).f_{1}\prime .G.{f^{\prime }}_{1}.(\gamma_{0}+\gamma_{1}) \end{array}\right\}dV$$

After integrating Equation (14) over the beam section and rewriting it in the matrix form, the variation of the strain energy can be computed as

(15) $$\delta \Pi =\int_{L}\left(\delta \omega^{T}R+\delta \gamma_{01}^{T}T_{01}\right)dx$$

where R and $T_{01}$ are given by

(16) $$\begin{array}{l} R=b\int_{z}\left[\begin{array}{cccc} D & f_{1}D & f_{2}D & g^{\prime }D\\ f_{1}D & f_{1}^{2}D & f_{1}f_{2}D & f_{1}g^{\prime }D\\ f_{2}D & f_{1}f_{2}D & f_{2}^{2}D & f_{2}g^{\prime }D\\ g^{\prime }D & g^{\prime }f_{1}D & g^{\prime }f_{2}D & g^{\prime }g^{\prime }D \end{array}\right]\left[\begin{array}{l} \varepsilon^{0}\\ \varepsilon^{1}\\ \varepsilon^{2}\\ \varepsilon^{3} \end{array}\right]dz\;,\;\\ T_{01}=b\int_{z}f_{1}\prime \tau dz=b\int_{z}{\left(f_{1}\prime \right)}^{2}G\gamma_{01}dz \end{array}$$

and

(17) $$\delta \omega =\left[\begin{array}{l} \delta \varepsilon^{0}\\ \delta \varepsilon^{1}\\ \delta \varepsilon^{2}\\ \delta \varepsilon^{3} \end{array}\right]\;,\;\delta \gamma_{01}=\delta \varphi_{,x}+\delta w_{,x}+\delta \beta$$

where

(18) $$\delta \varepsilon^{0}=\left\{\begin{array}{l} \delta u_{,x}\\ 0 \end{array}\right\},\;\delta \varepsilon^{1}=\left\{\begin{array}{l} \delta \beta_{,x}\\ 0 \end{array}\right\},\;\delta \varepsilon^{2}=\left\{\begin{array}{l} \delta \alpha_{,x}\\ 0 \end{array}\right\},\;\delta \varepsilon^{3}=\left\{\begin{array}{l} 0\\ \delta \varphi \end{array}\right\}$$

As consequence, Equation (16) can be rewritten as

(19) $$R=H\omega \;;\;T_{01}=H_{s}\gamma_{01}$$

where

(20) $$H=b\int_{z}\left[\begin{array}{cccc} D & f_{1}D & f_{2}D & g^{\prime }D\\ f_{1}D & f_{1}^{2}D & f_{1}f_{2}D & f_{1}g^{\prime }D\\ f_{2}D & f_{1}f_{2}D & f_{2}^{2}D & f_{2}g^{\prime }D\\ g^{\prime }D & g^{\prime }f_{1}D & g^{\prime }f_{2}D & g^{\prime }g^{\prime }D \end{array}\right]dz$$

(21) $$H_{s}=b\int_{z}{\left(f_{1}\prime \right)}^{2}Gdz$$

In the current work, a two-node beam element is considered, each node includes five degrees of freedom. The vector of displacement of node *i*-*th* is

(22) $$\left\{d_{i}\right\}={\left[\begin{array}{lllll} u_{i} & w_{i} & \varphi_{i} & \beta_{i} & \alpha_{i} \end{array}\right]}^{T}$$

The nodal displacement vector of the proposed beam element, U, is defined by

(23) $$U={\left[\begin{array}{llllllllll} u_{1} & w_{1} & \varphi_{1} & \beta_{1} & \alpha_{1} & u_{2} & w_{2} & \varphi_{2} & \beta_{2} & \alpha_{2} \end{array}\right]}^{T}$$

The isoparametric geometry on two nodes of new beam element and the nodal variables are given by

(24) $$\{\begin{array}{cc} x=N_{1}x_{1}+N_{2}x_{2} & u=N_{1}u_{1}+N_{2}u_{2}\\ w=N_{1}w_{1}+N_{2}w_{2} & \varphi =N_{1}\varphi_{1}+N_{2}\varphi_{2}\\ \beta =N_{1}\beta_{1}+N_{2}\beta_{2} & \alpha =N_{1}\alpha_{1}+N_{2}\alpha_{2} \end{array}$$

For the mixed finite element formulation, the authors approve a quadratic interpolation for α, β with parameters $\alpha_{m},\;\beta_{m}$ and a constant shear resultant $T_{01},$ which will be eliminated later.

(25) $$\begin{array}{l} \alpha =N_{1}\alpha_{1}+N_{2}\alpha_{2}+N_{m}\alpha_{m}\\ \beta =N_{1}\beta_{1}+N_{2}\beta_{2}+N_{m}\beta_{m} \end{array}$$

(26) $$T_{01}=T_{0}-F(x),\;\delta T_{01}=\delta T_{0},\;F(x)=\int_{0}^{x}q(s)ds\;$$

in which, the shape functions $N_{1},\;N_{2}$ and $N_{m}$ are defined as follows

(27) $$\begin{array}{ccc} N_{1}=\frac{1-\xi }{2}, & N_{2}=\frac{1+\xi }{2}, & N_{m}=N_{1}N_{2}=\frac{1-\xi^{2}}{4} \end{array}$$

where

(28) $$\xi =\frac{2x-L}{L},\;d\xi =\frac{2}{L}dx,\;dx=\frac{L}{2}d\xi$$

The first equation in Equation (8) is imposed in integral form as following

(29) $$\int_{0}^{L}\gamma_{2}dx=\int_{0}^{L}\left(w_{,x}+\alpha \right)dx=0$$

Substitute α and w from Equations (24) and (25) into Equation (29), the parameter $\alpha_{m}$ can be deduced as

(30) $$\alpha_{m}=\frac{6}{L}\left(w_{1}-w_{2}-\frac{L}{2}\left(\alpha_{1}+\alpha_{2}\right)\right)=B_{f0}U$$

where

(31) $$B_{f0}=\left[\begin{array}{cccc} \begin{array}{cc} 0 & \begin{array}{cc} \frac{6}{L} & 0 \end{array} \end{array} & \begin{array}{cc} 0 & -3 \end{array} & \begin{array}{cc} 0 & \frac{-6}{L} \end{array} & \begin{array}{ccc} 0 & 0 & -3 \end{array} \end{array}\right]$$

Substitute Equations (24) and (30), into Equation (17), the strain variation vector, δω, can be obtained as

(32) $$\delta \omega =\left[\begin{array}{l} \delta \varepsilon^{0}\\ \delta \varepsilon^{1}\\ \delta \varepsilon^{2}\\ \delta \varepsilon^{3} \end{array}\right]$$

(33) $$\delta \varepsilon^{0}=\left\{\begin{array}{l} \delta u_{,x}\\ 0 \end{array}\right\},\;\delta \varepsilon^{1}=\left\{\begin{array}{l} \delta \beta_{,x}\\ 0 \end{array}\right\},\;\delta \varepsilon^{2}=\left\{\begin{array}{l} \delta \alpha_{,x}\\ 0 \end{array}\right\},\;\delta \varepsilon^{3}=\left\{\begin{array}{l} 0\\ \delta \varphi \end{array}\right\}$$

Using Equation (30), the variable α becomes

(34) $$\alpha =\sum N_{i}\alpha_{i}+N_{m}\alpha_{m}=\sum N_{i}\alpha_{i}+N_{m}\left(B_{f0}U\right)$$

Then

(35) $$\delta \varepsilon^{2}=\left\{\begin{array}{l} \delta \alpha_{,x}\\ 0 \end{array}\right\}=\left\{\begin{array}{l} \sum N_{i,x}\delta \alpha_{i}\\ 0 \end{array}\right\}+\left\{\begin{array}{l} N_{m,x}B_{f0}\delta U\\ 0 \end{array}\right\}$$

Using Equations (33) and (35), the strain variation Equation (32) can be expressed as follows

(36) $$\delta \omega =B\delta U=\left(B_{mf}+B_{f}\right)\delta U$$

where

(37) $$B_{mf}=\left[\begin{array}{cc} B_{mf}^{1} & B_{mf}^{2} \end{array}\right]$$

(38) $$B_{mf}^{1}=\left[\begin{array}{lllll} N_{1,x} & 0 & 0 & 0 & 0\\ 0 & 0 & 0 & 0 & 0\\ 0 & 0 & 0 & N_{1,x} & 0\\ 0 & 0 & 0 & 0 & 0\\ 0 & 0 & 0 & 0 & N_{1,x}\\ 0 & 0 & 0 & 0 & 0\\ 0 & 0 & 0 & 0 & 0\\ 0 & 0 & N_{1} & 0 & 0 \end{array}\right],\;B_{f}=\left[\begin{array}{l} 0_{1\times 10}\\ 0_{1\times 10}\\ 0_{1\times 10}\\ 0_{1\times 10}\\ N_{m,x}B_{f0}\\ 0_{1\times 10}\\ 0_{1\times 10}\\ 0_{1\times 10} \end{array}\right]$$

The shear strain variation $\delta \gamma_{01}$ can be obtained as

(39) $$\delta \gamma_{01}=\delta \varphi_{,x}+\delta w_{,x}+\delta \beta$$

Rewrite Equation (39) into the matrix form as below

(40) $$\delta \gamma_{01}=B_{s}\delta U$$

where

(41) $$B_{s}=\left[\begin{array}{llllllllll} 0 & N_{1,x} & N_{1,x} & N_{1} & 0 & 0 & N_{2,x} & N_{2,x} & N_{2} & 0 \end{array}\right]$$

Using Equation (25) the strain fields $\delta \varepsilon ,\;\delta \gamma_{01}$ become

(42) $$\delta \omega =B\delta U+B_{m}\delta \beta_{m}\;,\;B_{m}={\left[\begin{array}{ccc} \begin{array}{cc} 0 & 0 \end{array} & N_{m,x} & \begin{array}{lllll} 0 & 0 & 0 & 0 & 0 \end{array} \end{array}\right]}^{T}$$

(43) $$\delta \gamma_{01}=B_{s}\delta U+N_{m}\delta \beta_{m}$$

The variation of strain energy Equation (15) becomes

(44) $$\delta \Pi =\int_{0}^{L}\left(\delta \omega^{T}R+\delta \gamma_{01}T_{01}+\delta {Τ}_{01}\left(\gamma_{01}-\frac{T_{01}}{H_{s}}\right)\right)dx$$

Substituting $T_{01}=T_{0}-F\;,\;\delta T_{01}=\delta T_{0}$ into Equation (44) we get

(45) $$\delta \Pi =\int_{0}^{L}\left(\delta \omega^{T}R+\delta \gamma_{01}\left(T_{0}-F\right)+\delta {Τ}_{0}\left(\gamma_{01}-\frac{T_{0}-F}{H_{s}}\right)\right)dx$$

(46) $$\begin{array}{l} \delta \Pi =\int_{0}^{L}(\delta U^{T}B^{T}HBU+\delta U^{T}B^{T}HB_{m}\beta_{m}+\\ \delta \beta_{m}B_{\beta m}^{T}HBU+\delta \beta_{m}B_{\beta m}^{T}HB_{\beta m}\beta_{m}+\\ \delta U^{T}B_{s}{}^{T}T_{0}-\delta U^{T}B_{s}{}^{T}F+\delta \beta_{m}{}^{T}N_{m}T_{0}-\delta \beta_{m}{}^{T}N_{m}F+\\ \delta T_{0}B_{s}U+\delta T_{0}N_{m}\beta_{m}-\delta T_{0}\frac{1}{H_{s}}T_{0}-\delta T_{0}\frac{-1}{H_{s}}F)dx \end{array}$$

Rewritten Equation (46) in the matrix form

(47) $$\delta \Pi =\int_{0}^{L}\left\{\left[\begin{array}{ccc} \delta U^{T} & \delta T_{0} & \delta \beta_{m} \end{array}\right]\left(\left[\begin{array}{ccc} K_{uu} & K_{uT} & K_{u\beta }\\ K_{uT}^{T} & K_{TT} & K_{T\beta }\\ K_{u\beta }^{T} & K_{T\beta } & K_{\beta \beta } \end{array}\right]\left[\begin{array}{c} U\\ T_{0}\\ \beta_{m} \end{array}\right]-\left[\begin{array}{l} f_{u}\\ f_{T}\\ f_{\beta } \end{array}\right]\right)\right\}dx$$

where $K_{uu},\;K_{uT},\;K_{u\beta },\;K_{TT},\;K_{T\beta },\;K_{\beta \beta },\;f_{u},\;f_{T}$ and $f_{\beta }$ are calculated as follows

(48) $$K_{uu}=\int_{-1}^{1}B^{T}HB\frac{L}{2}d\xi =\int_{-1}^{1}{\left(B_{mf}+B_{f}\right)}^{T}H\left(B_{mf}+B_{f}\right)\frac{L}{2}d\xi$$

(49) $$K_{uT}=\int_{-1}^{1}B_{s}^{T}\frac{L}{2}d\xi$$

(50) $$K_{u\beta }=\int_{-1}^{1}B^{T}HB_{\beta m}\frac{L}{2}d\xi$$

(51) $$K_{TT}=-\int_{-1}^{1}\frac{1}{H_{s}}\frac{L}{2}d\xi$$

(52) $$K_{T\beta }=\int_{-1}^{1}N_{m}\frac{L}{2}d\xi$$

(53) $$K_{\beta \beta }=\int_{-1}^{1}B_{\beta m}^{T}HB_{\beta m}\frac{L}{2}d\xi$$

(54) $$f_{u}=\int_{0}^{L}N_{w}^{T}qdx+\int_{0}^{L}B_{s}^{T}Fdx$$

(55) $$f_{T}=\frac{-1}{H_{s}}\int_{0}^{L}F(x)dx$$

(56) $$f_{\beta }=\int_{0}^{L}N_{m}(x)F(x)dx$$

where

(57) $$N_{w}=\left[\begin{array}{llllllllll} 0 & N_{1} & 0 & 0 & 0 & 0 & N_{2} & 0 & 0 & 0 \end{array}\right]$$

The parameter $T_{0}$ and rotation $\beta_{m}$ are then removed in two steps as below.

**Step 1**: eliminate rotation parameter $\beta_{m}$

(58) $$\beta_{m}=\frac{1}{K_{\beta \beta }}\left(f_{b}-K_{u\beta }^{T}U-K_{T\beta }T_{0}\right)$$

Substitute Equation (58) into Equation (47), we get

(59) $$\delta \Pi =\int_{0}^{L}\left\{\left[\begin{array}{cc} \delta U^{T} & \delta T_{0} \end{array}\right]\left(\left[\begin{array}{cc} K_{11} & K_{12}\\ K_{12}^{T} & K_{22} \end{array}\right]\left[\begin{array}{l} U\\ T_{0} \end{array}\right]-\left[\begin{array}{l} f_{1}\\ f_{2} \end{array}\right]\right)\right\}dx$$

where $K_{11},\;K_{12},\;K_{22},\;f_{1}$ and $f_{2}$ are expressed as

(60) $$K_{11}=K_{uu}-K_{u\beta }K_{\beta \beta }^{-1}K_{u\beta }^{T}$$

(61) $$K_{12}=K_{uT}-K_{u\beta }K_{\beta \beta }^{-1}K_{T\beta }$$

(62) $$K_{22}=K_{TT}-K_{T\beta }K_{\beta \beta }^{-1}K_{T\beta }$$

(63) $$f_{1}=f_{u}-K_{u\beta }K_{\beta \beta }^{-1}f_{\beta }$$

(64) $$f_{2}=f_{T}-K_{T\beta }K_{\beta \beta }^{-1}f_{\beta }$$

**Step 2**: eliminate the stress resultant constant $T_{0}$

(65) $$T_{0}=K_{22}^{-1}\left(f_{2}-K_{12}^{T}U\right)$$

Substitute Equation (65) into Equation (59), the expression of the variation of strain energy is manifested as

(66) $$\delta \Pi =\delta U^{T}\left(K_{11}-K_{12}K_{22}^{-1}K_{12}^{T}\right)U-\delta U^{T}\left(f_{1}-K_{12}K_{22}^{-1}f_{2}\right)$$

The stiffness matrix of new beam element K is now calculated as

(67) $$K=K_{11}-K_{12}K_{22}^{-1}K_{12}^{T}$$

The nodal load vector of the beam element is expressed as

(68) $$f=f_{1}-K_{12}K_{22}^{-1}f_{2}$$

## 5. Numerical Results and Discussion

### 5.1. Convergence Study

In this subsection, some examples are given to determine the convergence rate of the proposed beam element. A homogeneous beam with the width b=1 m, Young’s modulus E=29000 Pa, Poisson’s ratio ν=0.3 as in the work of Heyliger [40] is considered here.

Firstly, a cantilever beam subject to a concentrated load P=100 N at the free end side is considered. Secondly, a simply supported beam under a uniform load q=10 N/m is investigated. The numerical results for some cases of L/h ratios and number of elements are shown in Table 1 and Table 2. The numerical results are compared with results of Heyliger [40] using two-node beam $C^{1}$ continuous formulation element based on HSDT. The comparison shows that the new beam element has an excellent convergence rate. Although the new proposed beam element uses linear shape functions, it has a better convergence rate than that of the beam element using higher-order shape function, thus it costs less effort and time of computation.

### 5.2. Validation Study

Continuously, to confirm the accuracy of the proposed beam element, the static bending of an FG beam subjected to a uniform load q is investigated. The FG beams made of two components, which are Aluminum and Alumina. The material properties of Aluminum and Alumina are $E_{m}=70\;\mathrm{GPa},$ $E_{c}=380\;\mathrm{GPa},$ $\nu_{m}=0.3,$ $\nu_{c}=0.3$. Two cases of slenderness ratios L/h=5 and L/h=20 of the FG beams are considered. The displacements and stresses are calculated in the normalized form as.

For simply-simply (SS) and clamped-clamped (CC) supported beams

(69) $$w^{*}=\frac{100E_{m}h^{3}}{qL^{4}}w\left(\frac{L}{2},0\right)$$

For clamped-free (CF) supported beams

(70) $$w^{*}=\frac{100E_{m}h^{3}}{qL^{4}}w\left(L,0\right)$$

For axial, normal and shear stresses

(71) $$\sigma_{x}^{*}=\frac{h}{qL}\sigma_{x}\left(\frac{L}{2},\frac{h}{2}\right),\;\sigma_{z}^{*}=\frac{h}{qL}\sigma_{z}\left(\frac{L}{2},\frac{h}{2}\right),\;\sigma_{xz}^{*}=\frac{h}{qL}\sigma_{xz}\left(0,0\right)$$

The numerical results of dimensionless vertical displacement, normal stress and axial stress of FG beam using proposed beam are compared with the results of Li et al. [16] and Vo et al. [20] using analytical and finite element methods, which are given in Table 3, Table 4, Table 5, Table 6 and Table 7. The results in these tables show that the solutions from the present theory are very close with the results from HSDT of Li et al. [16] and Quasi-3D solutions of Vo [20] for different values of the power-law index, aspect ratio, and boundary conditions.

**Figure 2:**
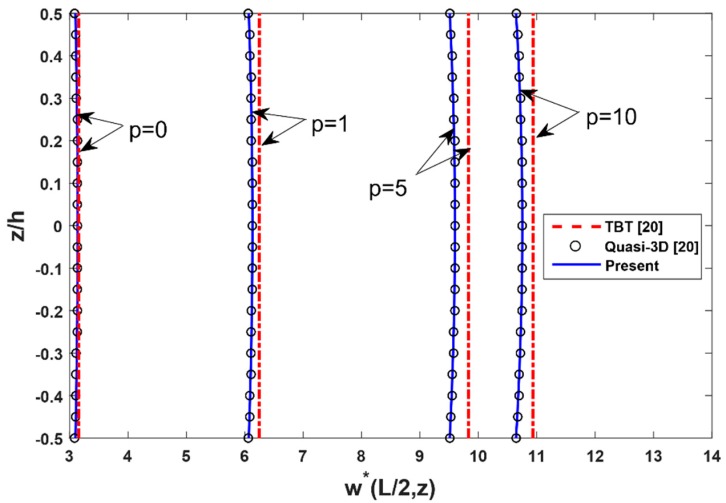
The comparison of the nondimensional transverse displacement $w^{*}(L/2,z)$ across the depth of FG SS beams subjected to a uniform load with L/h=5.

Figure 2 displays the comparison of the distribution of vertical displacement along with the depth of the FG beam with different values of the power-law index. It can be observed that the vertical displacements are variable across the thickness of the beam and they are in good agreement with published results of Li et al. [16] and Vo [20].

**Figure 3:**
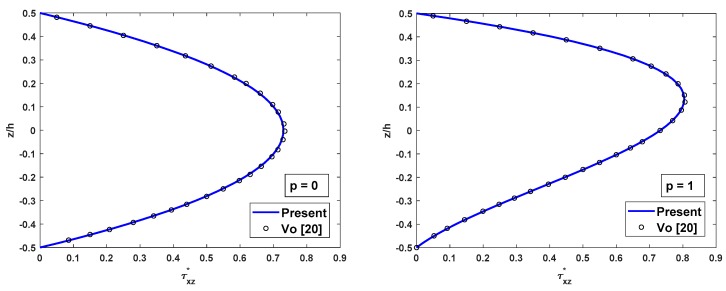

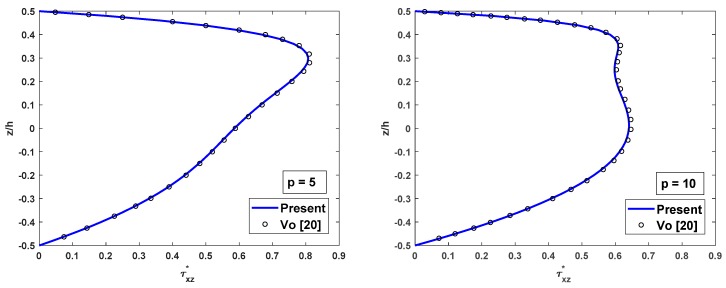
The comparison of the nondimensional shear stress $\tau_{xz}^{*}$ across the depth of FG SS beams subjected to a uniform load for different values of p with L/h=5.

**Figure 4:**
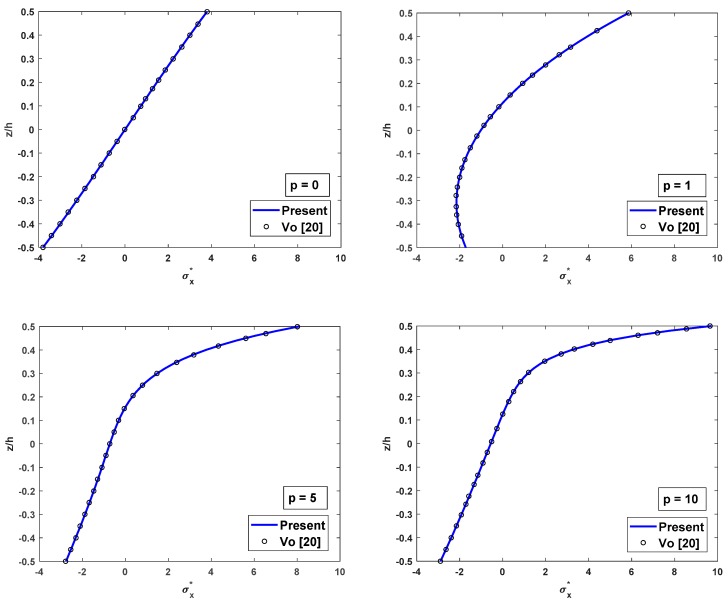
The comparison of the nondimensional axial stress $\sigma_{x}^{*}$ across the depth of FG SS beams subjected to a uniform load for different values of p with L/h=5.

The distributions of shear stress and axial stress along the depth of FG beam are compared with results of Li et al. [16] and Vo et al. [20] as in Figure 3 and Figure 4. According to Figure 3, the shear stress distribution is parabolic along with the thickness and asymmetric for the FG beams. From Figure 4, the axial stress variation is not linear across the thickness of the FG beam, and its variation is linear across the thickness for isotropic (full ceramic or full metal) beams only. In general, the values of the axial stress do not equal to zeros at the mid-plane of the FG beams. Both shear stress variation and axial stress variation through the thickness of the FG beam using the proposed beam element are in remarkable agreement with those of Vo [20] using Quasi-3D theory.

According to the comparison, the results of the proposed beam element are very close actual adjacent to the Li et al. [16] and Vo et al. [20] solutions. Therefore, the new beam element can be applied to analyze FG beams.

### 5.3. Static Behaviour of FG Beams

In this subsection, an FG beam which is produced of Aluminum and Zirconium dioxide ($Al/ZrO_{2}$) under uniform distribution load q is investigated using proposed beam element. Various power-law indexes, slenderness ratios and boundary conditions are considered. The material properties of Al are $E_{m}=70\;\mathrm{GPa},$ $\nu_{m}=0.3,$ and the material properties of $ZrO_{2}$ are $E_{c}=200\;\mathrm{GPa},$ $\nu_{c}=0.3$. The non-dimensional formulas are applied as follows

(72) $$w*=\frac{100h^{3}E_{m}}{qL^{4}}w,\sigma_{x}*=\frac{h}{qL}\sigma_{x},\sigma_{z}*=\frac{h}{qL}\sigma_{z},\sigma_{xz}*=\frac{h}{qL}\sigma_{xz}.$$

In this study, some cases of boundary conditions are considered.

For the simply-simply supported (SS) beam: u=w=0 at x=0, L;

For the clamped-clamped supported (CC) beam: u=w=α=β=0 at x=0, L;

For the clamped-simple supported (CS) beam: u=w=α=β=0 at x=0,  and u=w=0 at x=L;

For the clamped-free supported (CF) beam: u=w=α=β=0 at x=0.

**Figure 5:**
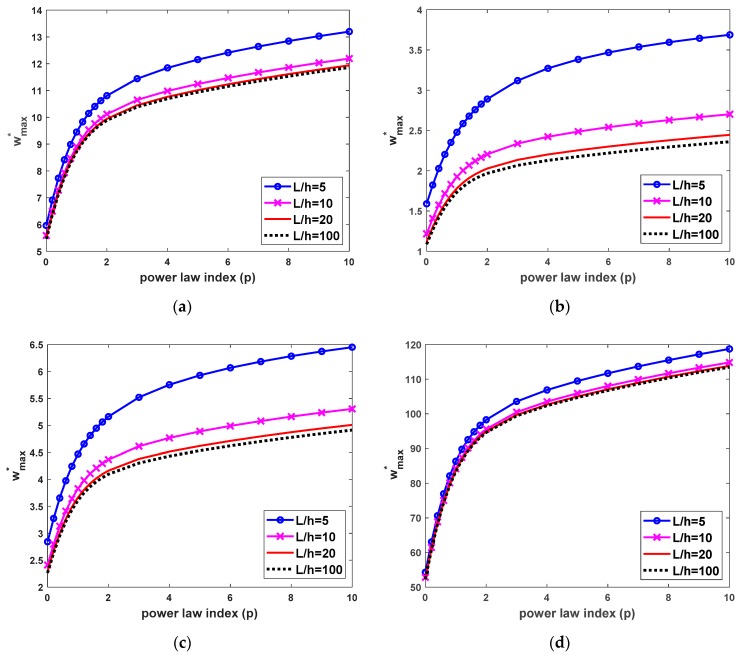
Nondimensional maximum transverse deflection $w_{\max}^{*}$ depends on the power-law index and length-to-thickness ratio of FG beams subjected to a uniform load, (**a**) SS beams, (**b**) CC beams, (**c**) CS beams and (**d**) CF beams.

**Figure 6:**
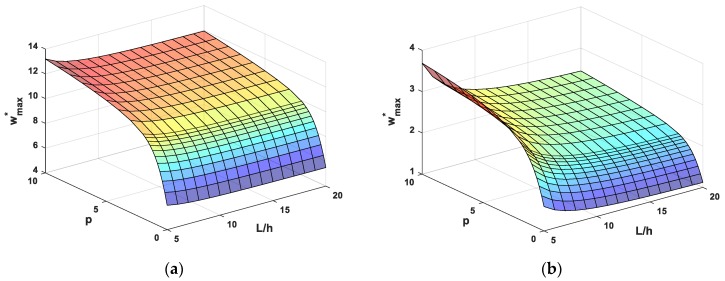

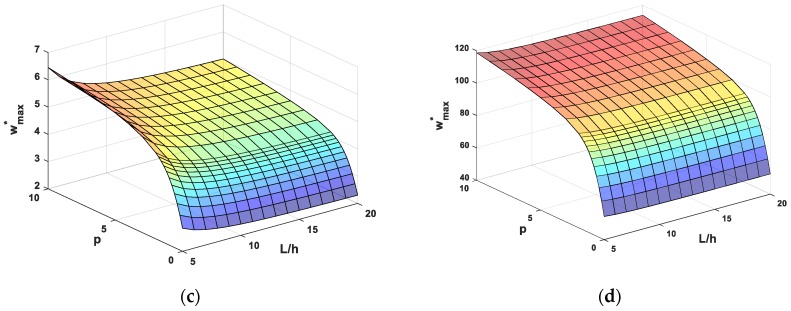
Nondimensional maximum transverse deflection $w_{\max}^{*}$ as a function of the power-law index and length-to-thickness ratio of FG beams subjected to a uniform load, (**a**) SS beams, (**b**) CC beams, (**c**) CS beams and (**d**) CF beams.

The numerical results for bending behaviors of FG beams under uniform load are shown in Table 8, Table 9, Table 10 and Table 11 and Figure 5 and Figure 6. Table 8 and Figure 5 shows the nondimensional maximum vertical displacement of FG beams for some cases of boundary conditions, power-law index and different values of the length-to-height ratio. To show more clearly the effect of the power-law index and slenderness ratio, Figure 6 shows the dependence of nondimensional maximum vertical displacement of FG beams on the continuous transformation of the power-law index and slenderness ratio. It shows that the deflection of FG beams increases when increasing the power-law index. This can be explained that increasing the value of the power-law index leads to an increase in the component of metal in FGM, so that the FG beam becomes more flexible. Furthermore, it can be observed that the nondimensional maximum vertical displacement depends not only on the power law index, slenderness ratio but also boundary conditions, which is more pronounced for CC and CS beams than SS and CF beams Furthermore, it can be observed that the nondimensional maximum vertical displacement depends on power-law distribution index, boundary conditions and the length-to-thickness ratio. In addition, boundary conditions have more strongly effects on the deflection of CC and CS beams than those of SS and CF beams.

**Figure 7:**
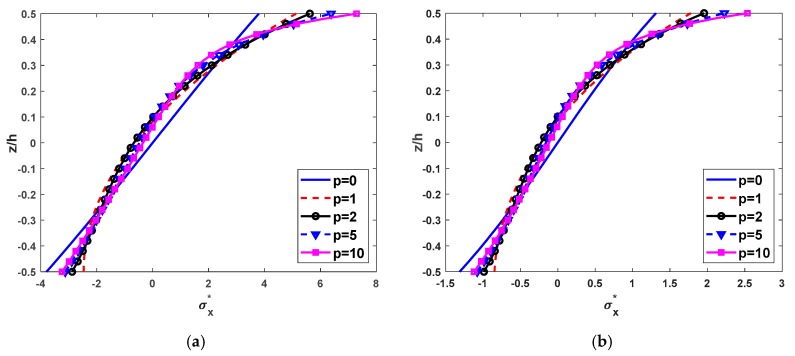
Nondimensional axial stress $\sigma_{x}^{*}(L/2,z)$ through the thickness of FG beams subjected to a uniform load with L/h=5, (**a**) SS beams and (**b**) CC beams.

Table 9 shows the nondimensional axial stress $\sigma_{x}^{*}(L/2,\;h/2)$ of FG beams subjected to a uniform load depends on some parameters and boundary conditions. Table 7 and Figure 7 present the distributions of nondimensional axial stress along with the depth of the SS and CC FG beams for different values of the power-law distribution index. The most significant aspect of this figure is that the axial stress distribution of FG beams is much more different from those of isotropic beams. As seen from Table 7 and Figure 7, the axial stress variation is not linear along with the thickness of the FG beams, the tensile stresses at the top are maximum. The values of the axial stresses do not equal to zeros at the mid-plane of the FG beams. This indicates that the neutral plane of the FG beams does not appear at the mid-plane, it is near the top face of the FG beams. This can be explained by the variation of the modulus of elasticity across the depth of the FG beams.

**Figure 8:**
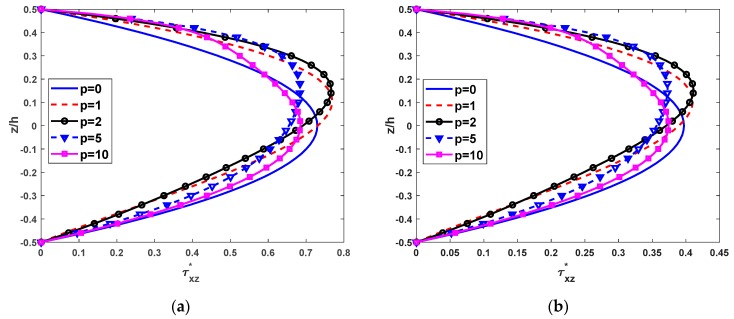
Nondimensional shear stress $\tau_{xz}^{*}(0,z)$ across the depth of FG beams subjected to a uniform load with L/h=5, (**a**) SS beams and (**b**) CC beams.

The non-dimensional shear stress distributions across the thickness of the beams made of FGM with different values of the power-law distribution index and some cases of boundary conditions are presented in Figure 8. The shear stresses of the full ceramic (isotropic) beams are symmetric about the mid-plane of the beams. In addition, the shear stress distributions are greatly influenced by the power-law index. In addition, Figure 8 shows the great dependence of the shear stress distribution on the power-law index.

The non-dimensional normal stresses of the FG beams under uniform distribution load are shown in Table 11, which highlight the effect of thickness stretching on bending behaviors of FG beam from Quasi-3D theory. Due to the thickness stretching effect, the vertical displacement obtained from present Quasi-3D theory is smaller than those of HSDT and FSDT.

**Figure 9:**
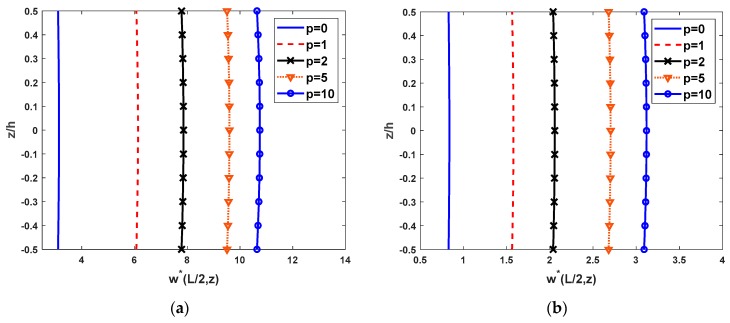
The distribution across the thickness of nondimensional vertical displacement of FG beam subjected to a uniform load with L/h=5, (**a**) SS beams and (**b**) CC beams.

The variation of the vertical displacement through the thickness of the FG beam for SS and CC boundary conditions are shown in Figure 9. According to Figure 9, the difference among the present Quasi-3D theory and other HSDT or FSDT is meaningful for thickness stretching. In this present Quasi-3D theory, the vertical displacement is not constant through the thickness of the beams as in HSDT and FSDT.

**Figure 10:**
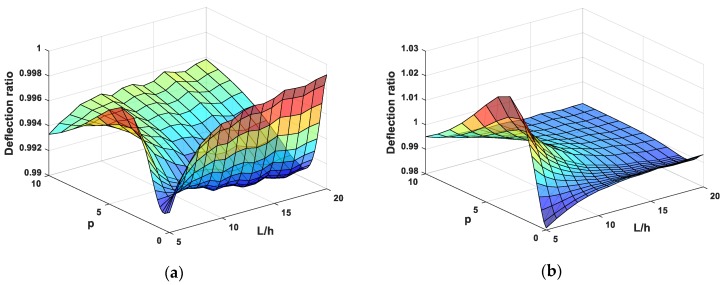
The deflection ratio of FG beams subjected to uniform load, (**a**) SS beams and (**b**) CC beams.

Finally, to show more obviously the influence of normal deformation on the deflection of FG beams, we suggest the deflection ratio which is well-defined as the fraction of transverse displacement obtained by present Quasi-3D beam theory to that calculated by HSDT. The effect of normal deformation on the deflection of SS and CC supported FG beams is exhibited in Figure 10 for different values of power-law distribution index and slenderness ratio. Figure 10 shows that the deflection ratio is almost smaller than unity. It shows that the deflection will be decreased when the normal deformation effect is included. In the case of CC beams, there is a range of power-law index and slenderness ratio that causes the deflection ratio to be greater than unity, which represents that the normal deformation has more affect strongly than bending and shear deformation in this case.

## 6. Conclusions

In this paper, a new efficient Quasi-3D beam element was developed for static bending analysis of FG beams. Using mixed formulation, only $C^{0}$ continuous shape functions are required for finite element formulation of the new beam element. In addition, the new beam element presents the excellent results of displacement and stress even for a coarse mesh. Therefore, the proposed beam element costs less effort and time of computation than those using higher order shape functions, consequently, it can be widely applied for complex structural analysis. The shear stresses vary parabolically across the thickness of the FG beam, and equal to zeros at two free surfaces of beams, so it does not need any shear correction factors. The new beam element includes shear deformation and normal deformation. Effect of normal deformation is significant, and it should be considered in the static bending analysis of FG beams, especially for medium and very thick FG beams. The numerical results of the FG beams using the proposed beam element are in good agreement with other published results. The new beam element is accurate and efficient for bending behavior of FG beams. The influences of some parameters such as the power-law distribution index and length-to-thickness ratio are investigated.

## Figures and Tables

**Table 1:** Comparison of maximum displacement for cantilever beams.

Length	Height	Source	Number of Elements			
			N = 2	N = 4	N = 8	N = 16
160	12	[40]	30.838	32.368	32.742	32.823
		Present	31.566	32.191	32.509	32.666
80		[40]	3.9234	4.1105	4.1506	4.1567
		Present	3.9987	4.0772	4.1160	4.1317
40		[40]	0.52266	0.54249	0.54540	0.54588
		Present	0.52608	0.53570	0.53907	0.53880
12		[40]	0.023551	0.023741	0.023874	0.023931
		Present	0.022902	0.022347	0.021967	0.021840
160	1	[40]	52968.0	55616.0	56278.0	56444.0
		Present	54302.8	55380.8	55931.1	56206.2
80		[40]	6621.8	6952.9	7035.6	7056.3
		Present	6788.5	6923.3	6992.0	7026.4
40		[40]	828.15	869.53	879.87	882.44
		Present	848.88	865.73	874.33	878.62
12		[40]	22.513	23.627	23.897	23.953
		Present	23.036	23.492	23.723	23.836

**Table 2:** Comparison of maximum displacement for simply supported beams.

Length	Height	Source	Number of Elements			
			N = 2	N = 4	N = 8	N = 16
160	12	[40]	19.779	20.529	20.691	20.717
		Present	20.692	20.690	20.690	20.690
80		[40]	1.3011	1.3415	1.3478	1.3486
		Present	1.3425	1.3422	1.3421	1.3421
40		[40]	0.096033	0.097481	0.097670	0.097703
		Present	0.096067	0.096060	0.096022	0.096018
12		[40]	0.0022234	0.0022206	0.0022204	0.0022204
		Present	0.0018828	0.0019482	0.0019524	0.0019523
160	1	[40]	33549.0	34873.0	35205.0	35287.0
		Present	35302.9	35302.9	35302.9	35302.9
80		[40]	2097.7	2180.4	2201.1	2206.3
		Present	2207.0	2207.0	2207.0	2207.0
40		[40]	131.31	136.49	137.77	138.08
		Present	138.09	138.09	138.09	138.09
12		[40]	1.0860	1.1267	1.1351	1.1364
		Present	1.1347	1.1346	1.1346	1.1346

**Table 3:** The maximum nondimensional vertical displacement of FG SS beams.

L/h	Source	$\sigma_{z}$	p=0	p=1	p=2	p=5	p=10
5	Li et al. [16]	=0	3.1657	6.2599	8.0602	9.7802	10.8979
	Vo [20] (Navier)	≠0	3.1397	6.1338	7.8606	9.6037	10.7578
	Vo [20] (FEM)	≠0	3.1397	6.1334	7.8598	9.6030	10.7572
	Present	≠0	3.1388	6.1316	7.8570	9.5992	10.7526
20	Li et al. [16]	=0	2.8962	5.8049	7.4415	8.8151	9.6879
	Vo [20] (Navier)	≠0	2.8947	5.7201	7.2805	8.6479	9.5749
	Vo [20] (FEM)	≠0	2.8947	5.7197	7.2797	8.6471	9.5743
	Present	≠0	2.8938	5.7179	7.2770	8.6435	9.5698

**Table 4:** The nondimensional normal stress of FG SS beams.

L/h	Source	$\sigma_{z}$	p=0	p=1	p=2	p=5	p=10
5	Vo [20] (Navier)	≠0	0.1352	0.0670	0.0925	0.0180	−0.0181
	Vo [20] (FEM)	≠0	0.1352	0.0672	0.0927	0.0183	−0.0179
	Present	≠0	0.1351	0.0669	0.0924	0.0179	−0.0183
20	Vo [20] (Navier)	≠0	0.0337	−0.5880	−0.6269	−1.1698	−1.5572
	Vo [20] (FEM)	≠0	0.0338	−0.5874	−0.6261	−1.1690	−1.5560
	Present	≠0	0.0337	−0.5880	−0.6270	−1.1696	−1.5570

**Table 5:** The nondimensional axial stress of FG SS beams.

L/h	Source	$\sigma_{z}$	p=0	p=1	p=2	p=5	p=10
5	Li et al. [16]	=0	3.8020	5.8837	6.8812	8.1030	9.7063
	Vo [20] (Navier)	≠0	3.8005	5.8812	6.8818	8.1140	9.7164
	Vo [20] (FEM)	≠0	3.8020	5.8840	6.8860	8.1190	9.7220
	Present	≠0	3.7994	5.8793	6.8792	8.1101	9.7108
20	Li et al. [16]	=0	15.0130	23.2054	27.0989	31.8112	38.1372
	Vo [20] (Navier)	≠0	15.0125	23.2046	27.0988	31.8137	38.1395
	Vo [20] (FEM)	≠0	15.0200	23.2200	27.1100	31.8300	38.1600
	Present	≠0	15.0079	23.1970	27.0884	31.7987	38.1176

**Table 6:** The nondimensional shear stress of FG SS beams.

L/h	Source	$\sigma_{z}$	p=0	p=1	p=2	p=5	p=10
5	Li et al. [16]	=0	0.7500	0.7500	0.6787	0.5790	0.6436
	Vo [20] (Navier)	≠0	0.7233	0.7233	0.6622	0.5840	0.6396
	Vo [20] (FEM)	≠0	0.7291	0.7291	0.6661	0.5873	0.6439
	Present	≠0	0.7233	0.7233	0.6622	0.5839	0.6396
20	Li et al. [16]	=0	0.7500	0.7500	0.6787	0.5790	0.6436
	Vo [20] (Navier)	≠0	0.7432	0.7432	0.6809	0.6010	0.6583
	Vo [20] (FEM)	≠0	0.7466	0.7466	0.6776	0.6036	0.6675
	Present	≠0	0.7454	0.7457	0.6828	0.6022	0.6595

**Table 7:** The maximum nondimensional vertical displacement of FG CC and CF beams.

L/h	Boundary Condition	Source	p=0	p=1	p=2	p=5	p=10
5	CC	Vo [20]	0.8327	1.5722	2.0489	2.6929	3.1058
		Present	0.8367	1.5787	2.0568	2.7039	3.1193
	CF	Vo [20]	28.5524	56.2002	71.7295	86.1201	95.7582
		Present	28.5743	56.2359	71.7607	86.1492	95.7903
20	CC	Vo [20]	0.5894	1.1613	1.4811	1.7731	1.9694
		Present	0.5894	1.1612	1.4806	1.7726	1.9689
	CF	Vo [20]	27.6217	54.6285	69.5266	82.4836	91.2606
		Present	27.6087	54.6051	69.4911	82.4327	91.1965

**Table 8:** Nondimensional maximum vertical displacement of FG beams subjected to a uniform load.

Boundary Condition	p	L/h = 5	L/h = 10	L/h = 20	L/h = 100
SS	0	5.9637	5.5917	5.4983	5.4684
	1	9.4520	8.9008	8.7625	8.7182
	2	10.8090	10.1178	9.9444	9.8888
	5	12.1559	11.2427	11.0136	10.9402
	10	13.1998	12.1936	11.9411	11.8602
CC	0	1.5898	1.2166	1.1200	1.0877
	1	2.4783	1.9253	1.7823	1.7344
	2	2.8903	2.2046	2.0270	1.9676
	5	3.3827	2.4869	2.2545	2.1770
	10	3.6885	2.7014	2.4453	2.3599
CS	0	2.8431	2.4121	2.3013	2.2650
	1	4.4681	3.8293	3.6651	3.6114
	2	5.1625	4.3680	4.1635	4.0966
	5	5.9291	4.8883	4.6201	4.5323
	10	6.4522	5.3055	5.0099	4.9132
CF	0	54.2912	52.8297	52.4566	52.3199
	1	86.3563	84.1912	83.6384	83.4359
	2	98.2871	95.5870	94.8959	94.6463
	5	109.4815	105.9308	105.0199	104.6959
	10	118.7523	114.8445	113.8429	113.4858

**Table 9:** Nondimensional axial stress $\sigma_{x}^{*}(L/2,\;h/2)$ of FG beams subjected to a uniform load.

Boundary Condition	p	L/h = 5	L/h = 10	L/h = 20	L/h = 100
SS	0	3.7994	7.5229	15.0080	74.9791
	1	5.1277	10.1431	20.2300	101.0602
	2	5.6251	11.1138	22.1592	110.6870
	5	6.3879	12.6061	25.1275	125.5017
	10	7.2947	14.4105	28.7315	143.5145
CC	0	1.3158	2.5271	5.0069	24.9844
	1	1.7824	3.4099	6.7496	33.6740
	2	1.9604	3.7389	7.3948	36.8820
	5	2.2314	4.2440	8.3871	41.8189
	10	2.5409	4.8479	9.5884	47.8209
CS	0	1.9797	3.7881	7.4945	37.3702
	1	2.6694	5.1069	10.1027	50.3710
	2	2.9383	5.6014	11.0697	55.1718
	5	3.3561	6.3638	12.5573	62.5539
	10	3.8300	7.2728	14.3568	71.5291
CF	0	−3.7207	−7.5172	−15.0722	−75.4217
	1	−5.0080	−10.1282	−20.3126	−101.6530
	2	−5.4757	−11.0879	−22.2441	−111.3295
	5	−6.1993	−12.5682	−25.2212	−126.2416
	10	−7.0995	−14.3779	−28.8453	−144.3695

**Table 10:** Nondimensional shear stress $\sigma_{xz}^{*}(0,0)$ of the FG beams subjected to a uniform load.

Boundary Condition	p	L/h = 5	L/h = 10	L/h = 20	L/h = 100
SS	0	0.7233	0.7370	0.7454	0.8112
	1	0.7233	0.7370	0.7455	0.8143
	2	0.6857	0.6989	0.7068	0.7660
	5	0.6513	0.6640	0.6714	0.7199
	10	0.6821	0.6954	0.7031	0.7536
CC	0	0.3330	0.1316	−0.2769	−4.4717
	1	0.3322	0.1283	−0.2900	−4.7991
	2	0.3060	0.1198	−0.2567	−4.1445
	5	0.2809	0.1137	−0.2160	−3.3285
	10	0.2937	0.1179	−0.2275	−3.4354
CS	0	0.3727	0.0596	−0.5617	−6.8939
	1	0.3710	0.0544	−0.5814	−7.3858
	2	0.3419	0.0525	−0.5203	−6.3910
	5	0.3148	0.0542	−0.4483	−5.1543
	10	0.3290	0.0550	−0.4717	−5.3222
CF	0	−0.2182	−1.5237	−4.0538	−29.8114
	1	−0.2231	−1.5435	−4.1329	−31.7882
	2	−0.2098	−1.4184	−3.7546	−27.6572
	5	−0.1921	−1.2813	−3.3351	−22.5588
	10	−0.2071	−1.3524	−3.5048	−23.3207

**Table 11:** Nondimensional normal stress $\sigma_{z}^{*}(L/2,\;h/2)$ of FG beams subjected to a uniform load.

Boundary Condition	p	L/h = 5	L/h = 10	L/h = 20	L/h = 100
SS	0	0.1351	0.0675	0.0337	0.0065
	1	0.0499	−0.2005	−0.5512	−2.9963
	2	0.0389	−0.2557	−0.6781	−3.6571
	5	0.0375	−0.3064	−0.8035	−4.3225
	10	0.0788	−0.2659	−0.7435	−4.0561
CC	0	0.1351	0.0675	0.0337	0.0065
	1	0.1501	−0.0001	−0.1504	−0.9923
	2	0.1611	−0.0112	−0.1890	−1.2118
	5	0.1820	−0.0174	−0.2255	−1.4326
	10	0.2145	0.0055	−0.2008	−1.3429
CS	0	0.1342	0.0657	0.0300	−0.0121
	1	0.1217	−0.0535	−0.2554	−1.5158
	2	0.1265	−0.0760	−0.3165	−1.8471
	5	0.1405	−0.0942	−0.3763	−2.1831
	10	0.1752	−0.0674	−0.3438	−2.0545
CF	0	0.1314	0.0601	0.0189	−0.0678
	1	0.3459	0.3915	0.6329	2.9244
	2	0.4008	0.4682	0.7696	3.5814
	5	0.4653	0.5492	0.9077	4.2336
	10	0.4791	0.5346	0.8575	3.9489

## References

[1] Anandakumar G., Kim J.H. (2010). On the modal behavior of a three-dimensional functionally graded cantilever beam: Poisson’s ratio and material sampling effects. Compos. Struct..

[2] Ebrahimi F., Mokhtari M. (2016). Free vibration analysis of a rotating Mori–Tanaka-based functionally graded beam via differential transformation method. Arab. J. Sci. Eng..

[3] Sankar B.V. (2001). An elasticity solution for functionally graded beams. Compos. Sci. Technol..

[4] Zenkour A.M. (2007). Benchmark trigonometric and 3-D elasticity solutions for an exponentially graded thick rectangular plate. Arch. Appl. Mech..

[5] Zhong Z., Yu T. (2007). Analytical solution of a cantilever functionally graded beam. Compos. Sci. Technol..

[6] Trinh L.C., Vo T.P., Thai H.T., Nguyen T.K. (2016). An analytical method for the vibration and buckling of functionally graded beams under mechanical and thermal loads. Compos. Part B.

[7] Kien N.D. (2014). Large displacement behaviour of tapered cantilever Euler–Bernoulli beams made of functionally graded material. Appl. Math. Comput..

[8] Lee J.W., Lee Y.J. (2016). Free vibration analysis of functionally graded Bernoulli-Euler beams using an exact transfer matrix expression. Int. J. Mech. Sci..

[9] Menaa R., Tounsi A., Mouaici F., Mechab I., Zidi M., Bedia E.I. (2012). Analytical solutions for static shear correction factor of functionally graded rectangular beams. Mech. Adv. Mater. Structu..

[10] Murin J., Aminbaghai M., Hrabovsky J., Kutis V., Kugler S. (2013). Modal analysis of the FGM beams with effect of the shear correction function. Compos. Part B.

[11] Nguyen T.K., Vo T.P., Thai H.T. (2013). Static and free vibration of axially loaded functionally graded beams based on the first-order shear deformation theory. Compos. Part B.

[12] Nam V.H., Vinh P.V., Chinh N.V., Thom D.V., Hong T.T. (2019). A new beam model for simulation of the mechanical behaviour of variable thickness functionally graded material beams based on modified first order shear deformation theory. Materials.

[13] Shi G. (2007). A new simple third-order shear deformation theory of plates. Int. J. Solids Struct..

[14] Kadoli R., Akhtar K., Ganesan N. (2008). Static analysis of functionally graded beams using higher order shear deformation theory. Appl. Math. Model..

[15] Benatta M.A., Mechab I., Tounsi A., Adda Bedia E.A. (2008). Static analysis of functionally graded short beams including warping and shear deformation effects. Comput. Mater. Sci..

[16] Li X.F., Wang B.L., Han J.C. (2010). A higher-order theory for static and dynamic analyses of functionally graded beams. Arch. Appl. Mech..

[17] Thai H.T., Vo T.P. (2012). Bending and free vibration of functionally graded beams using various higher-order shear deformation beam theories. Int. J. Mech. Sci..

[18] Vo T.P., Thai H.T., Nguyen T.K., Iman F. (2014). Static and vibration analysis of functionally graded beams using refined shear deformation theory. Meccanica.

[19] Tinh Q.B., Thom V.D., Lan H.T.T., Duc H.D., Satoyuki T., Dat T.P., Thien-An N.V., Yu T.T., Sohichi H. (2016). On the high temperature mechanical behaviors analysis of heated functionally graded plates using FEM and a new third-order shear deformation plate theory. Compos. Part B.

[20] Vo T.P., Thai H.T., Nguyen T.K., Iman F., Lee J. (2015). Static behaviour of functionally graded sandwich beams using a Quasi-3D theory. Compos. Part B.

[21] Neves A.M.A., Ferreira A.J.M., Carrera E., Roque C.M.C., Cinefra M., Jorge R.M.N., Soares C.M.M. (2012). A Quasi-3D sinusoidal shear deformation theory for the static and free vibration analysis of functionally graded plates. Compos. Part B.

[22] Neves A.M.A., Ferreira A.J.A., Carrera E., Cinefra M., Roque C.M.C., Jorge R.M.N., Soares C.M.M. (2012). A Quasi-3D hyperbolic shear deformation theory for the static and free vibration analysis of functionally graded plates. Compos. Struct..

[23] Hebali H., Tounsi A., Houari M.S.A., Bessain A., Adda Bedia E.A. (2014). New Quasi-3D hyperbolic shear deformation theory for the static and free vibration analysis of functionally graded plates. J. Eng. Mech..

[24] Mantari J.L., Soares C.G. (2012). Generalized hybrid Quasi-3D shear deformation theory for the static analysis of advanced composite plates. Compos. Struct..

[25] Mantari J.L., Soares C.G. (2014). Four-unknown Quasi-3D shear deformation theory for advanced composite plates. Compos. Struct..

[26] Thai H.T., Vo T.P., Bui T.Q., Nguyen T.K. (2014). A Quasi-3D hyperbolic shear deformation theory for functionally graded plates. Acta Mech..

[27] Fang W., Yu T., Lich L.V., Bui T.Q. (2019). Analysis of thick porous beams by a Quasi-3D theory and isogeometric analysis. Compos. Struct..

[28] Nguyen H.X., Nguyen T.N., Abdel-Wahab M., Bordas S.P.A., Hung N.X., Vo T.P. (2017). A refined Quasi-3D isogeometric analysis for functionally graded microplates based on the modifed couple stress theory. Comput. Methods Appl. Mech. Eng..

[29] Yu T., Zhang J., Hu H., Bui T.Q. (2019). A novel size-dependent Quasi-3D isogeometric beam model for two-directional FG microbeams analysis. Compos. Struct..

[30] Farzam-Rad S.A., Hassani B., Karamodin H. (2017). Isogeometric analysis of functionally graded plates using a new Quasi-3D shear deformation theory based on physical neutral surface. Compos. Part B.

[31] Tran L.V., Wahab M.A., Niiranen J. (2018). A six-variable Quasi-3D model for static analysis of laminated composite plates using isogeometric analysis. Int. Conf. Numer. Model. Eng..

[32] Carrera E. (2003). Theories and finite elements for multilayered plates and shells: A unified compact formulation with numerical assessment and benchmarking. Arch. Comput. Methods Eng..

[33] Carrera E., Petrolo M., Nali P. (2011). Unified formulation applied to free vibrations finite element analysis of beams with arbitrary section. Shock Vib..

[34] Cerrera E., Zozulya V.V. (2019). Carrera unified formulation (CUF) for the micropolar beams: Analytical solutions. Mech. Adv. Mater. Struct..

[35] Giunta G., Belouettar S., Carrera E. (2010). Analysis of FGM beams by means of classical and advanced theories. Mech. Adv. Mater. Struct..

[36] Filippi M., Carrera E., Zenkour A.M. (2015). Static analyses of FGM beams by various theories and finite elements. Compos. Part B.

[37] Chakraborty A., Gopalakrishnan S., Reddy J.N. (2003). A new beam finite element for the analysis of functionally graded materials. Int. J. Mech. Sci..

[38] Nguyen D.K., Nguyen Q.H., Tran T.T., Bui V.T. (2017). Vibration of bi-dimensional functionally graded Timoshenko beams excited by a moving load. Acta Mech..

[39] Khan A.A., Alam M.N., Rahman N., Wajid M. (2016). Finite element modelling for static and free vibration response of functionally graded beam. Lat. Am. J. Solids Struct..

[40] Heyliger P.R. (1988). A higher order beam finite element for bending and vibration problems. J. Sound Vib..

[41] Kapuria S., Bhattacharyya M., Kumar A.N. (2008). Bending and free vibration response of layered functionally graded beams: A theoretical model and its experimental validation. Compos. Struct..

[42] Vo T.P., Thai H.T., Nguyen T.K., Maheri A., Lee J. (2014). Finite element model for vibration and buckling of functionally graded sandwich beams based on a refined shear deformation theory. Eng. Struct..

[43] Moallemi-Oreh A., Karkon M. (2013). Finite element formulation for stability and free vibration analysis of Timoshenko beam. Adv. Acoust. Vib..

[44] Pascon J.P. (2016). Finite element analysis of flexible functionally graded beams with variable Poisson’s ratio. Eng. Comput..

[45] Yarasca J., Mantari J.L., Arciniega R.A. (2016). Hermite-Lagrangian finite element formulation to study functionally graded sandwich beams. Compos. Struct..

